# Clinical and laboratory predictors of influenza infection among individuals with influenza-like illness presenting to an urban Thai hospital over a five-year period

**DOI:** 10.1371/journal.pone.0193050

**Published:** 2018-03-07

**Authors:** Kathryn B. Anderson, Sriluck Simasathien, Veerachai Watanaveeradej, Alden L. Weg, Damon W. Ellison, Detchvijitr Suwanpakdee, Chonticha Klungthong, Thipwipha Phonpakobsin, Phirangkul Kerdpanich, Danabhand Phiboonbanakit, Robert V. Gibbons, Stefan Fernandez, Louis R. Macareo, In-Kyu Yoon, Richard G. Jarman

**Affiliations:** 1 Department of Virology, Armed Forces Research Institute of Medical Sciences, Bangkok, Thailand; 2 Department of Medicine, Division of General Internal Medicine, University of Minnesota, Minneapolis, Minnesota, United States of America; 3 Department of Pediatrics, Phramongkutklao Hospital, Bangkok, Thailand; 4 Viral Disease Branch, Walter Reed Army Institute of Research, Silver Spring, Maryland, United States of America; Defense Threat Reduction Agency, UNITED STATES

## Abstract

Early diagnosis of influenza infection maximizes the effectiveness of antiviral medicines. Here, we assess the ability for clinical characteristics and rapid influenza tests to predict PCR-confirmed influenza infection in a sentinel, cross-sectional study for influenza-like illness (ILI) in Thailand. Participants meeting criteria for acute ILI (fever > 38°C and cough or sore throat) were recruited from inpatient and outpatient departments in Bangkok, Thailand, from 2009–2014. The primary endpoint for the study was the occurrence of virologically-confirmed influenza infection (based upon detection of viral RNA by RT-PCR) among individuals presenting for care with ILI. Nasal and throat swabs were tested by rapid influenza test (QuickVue) and by RT-PCR. Vaccine effectiveness (VE) was calculated using the case test-negative method. Classification and Regression Tree (CART) analysis was used to predict influenza RT-PCR positivity based upon symptoms reported. We enrolled 4572 individuals with ILI; 32.7% had detectable influenza RNA by RT-PCR. Influenza cases were attributable to influenza B (38.6%), A(H1N1)pdm09 (35.1%), and A(H3N2) (26.3%) viruses. VE was highest against influenza A(H1N1)pdm09 virus and among adults. The most important symptoms for predicting influenza PCR-positivity among patients with ILI were cough, runny nose, chills, and body aches. The accuracy of the CART predictive model was 72.8%, with an NPV of 78.1% and a PPV of 59.7%. During epidemic periods, PPV improved to 68.5%. The PPV of the QuickVue assay relative to RT-PCR was 93.0% overall, with peak performance during epidemic periods and in the absence of oseltamivir treatment. Clinical criteria demonstrated poor predictive capability outside of epidemic periods while rapid tests were reasonably accurate and may provide an acceptable alternative to RT-PCR testing in resource-limited areas.

## Introduction

Infection with influenza viruses poses a significant public health threat globally, with a disproportionate impact in the developing world [1]. Southeast Asia is a particularly important region of interest for influenza epidemiology and ecology, with a high burden of disease and complex transmission patterns. Many areas experience year-round transmission, promoting the emergence and seeding of viruses into other regions.

As influenza vaccine programs build throughout Asia, important questions remain regarding regional epidemiology and vaccine effectiveness to inform local and global vaccination programs. In Thailand, influenza vaccination is recommended for health care workers, pregnant women, individuals with underlying comorbidities, young children, and the elderly. While vaccine coverage has been increasing, awareness of influenza vaccine recommendations and acceptance of vaccine administration remain a challenge in some target populations [2]. Both live and inactivated vaccines, and northern and southern hemisphere formulations, are available in Thailand, however, the inactivated formulation of the southern hemisphere vaccine predominates (Sriluck Simasathien, personal communication).

Neuraminidase inhibitors have been shown to decrease the duration of influenza-associated illness [3] and the occurrence of influenza-related complications [4]. However, oseltamivir remains profoundly underutilized, even in high-risk populations [5]. In the developing world, the low use of oseltamivir may be related in part to the unavailability or delayed receipt of confirmation for influenza infection. There is a need for more accessible diagnostic tools for influenza infection to facilitate the prompt administration of oseltamivir and to decrease unnecessary additional testing [6]. Rapid influenza diagnostic tests for influenza have been readily deployed across a range of clinical settings, however, the sensitivity of these tests has been shown to vary by influenza subtype [7], lineage [8], timing of specimen collection relative to symptom onset[9], and influenza viral load [10, 11]. An improved understanding of the performance characteristics and limitations of rapid tests is essential for appropriate clinical application and interpretation.

Multiple studies have sought to predict influenza from among influenza-like illness (ILI) cases based upon clinical data such as symptoms and exposure histories. Some clinical algorithms have demonstrated promising results and relatively high accuracy [12, 13], however, most have reported problematically low positive predictive values [14–17]. These clinical algorithms are likely affected by overlap in the clinical presentation of influenza and other respiratory pathogens but may offer some promise in identifying likely influenza cases in resource-limited settings.

In this manuscript, we explore epidemiologic, demographic, and clinical factors associated with influenza infection, influenza vaccination, and vaccine effectiveness in a cohort of individuals presenting with ILI in Thailand over a five-year period. We present a predictive model to discern influenza from non-influenza ILI based upon clinical symptoms and explore factors related to performance characteristics of the QuickVue rapid influenza diagnostic test. We seek to improve the predictive capability for influenza infection in regions with limited laboratory capabilities and to therefore improve clinical management and clinical outcomes.

## Materials and methods

### Location

The study was conducted at Phramongkutklao (PMK) hospital in Bangkok, Thailand. PMK is a 1200 bed hospital serving active and retired Royal Thai Army military personnel, their families, and other civilians. The majority of the patient population seen is civilian (60–70%). The hospital has inpatient and outpatient departments and sees both children and adults.

### Study population and study methods

Sentinel surveillance for ILI was established as part of the Armed Forces Research Institute of Medical Sciences (AFRIMS) influenza surveillance program at PMK hospital, initiated in 2009. The surveillance period for this manuscript was August 2009 through August 2014. The study is cross-sectional in design, with no specimen or data collection occurring after the initial visit. Participants were enrolled from inpatient and outpatient departments with the following inclusion criteria: fever > 38°C accompanied by cough or sore throat, age > = 6 months, presentation to PMK within 3 days of fever onset for outpatients and within 5 days of fever for inpatients, and provision of consent or assent for participation. Exclusion criteria were the presence of any immunocompromising condition or suspected tuberculosis.

Enrolled subjects provided demographic information and exposure information (recent travel, sick contacts, smoking history) as well as clinical information (presence / absence of specific symptoms, receipt of any medications for the illness prior to enrollment, presence of select comorbidities). Additionally, subjects were asked to report whether and when they had received an influenza vaccine within the last 12 months. A nasal swab was obtained for rapid influenza testing (QuickVue), which was performed onsite. An additional set of nasal and throat swabs were obtained, although two throat swabs were occasionally obtained from children per the parents’ or child’s wishes. Specimens were first tested for influenza A or B by reverse transcriptase polymerase chain reaction (RT-PCR); if positive for influenza A, they were further tested with primers specific for H1, H3 and H1 pandemic 2009 (pdm09). Primers and probes were designed by the US Centers for Disease Control and Prevention (CDC) [18, 19].

The study was approved by the Institutional Review Boards of the Royal Thai Army in Bangkok, Thailand, PMK hospital, and the Walter Reed Army Institute of Research (WRAIR). Written informed consent was obtained from all subjects.

### Statistical analysis

Bivariate analyses were conducted using χ^2^ testing for categorical and ANOVA for continuous variables. Age was divided into groupings of 0–4 years, 5–14 years, 15–59 years, and greater than 60 years. Comorbidities studied were those recommended by the US CDC to receive influenza vaccination [20]. Epidemic periods were defined as months where confirmed influenza cases (by RT-PCR) represented > = 25% of all ILI cases. Vaccine effectiveness (VE) was calculated using the ‘test negative’ method for estimating vaccine effectiveness in ILI investigations, where VE = (1- the odds ratio for disease, comparing vaccinated and unvaccinated populations) * 100%[21]. VE for individual influenza subtypes was calculated excluding influenza positive cases due to other serotypes from the comparison group. Data on influenza lineages circulating in Thailand during the study period were derived from the Thai National Influenza Center and based upon hemaggulatination inhibition and sequencing results [22].

Classification and regression tree (CART) analysis was used to develop a predictive model of influenza PCR-positivity among ILI cases based upon symptoms reported. All symptom variables (S1 Table) and age were incorporated as binary variables. The initial tree was obtained by recursive partitioning using the rpart package in R, which identifies the initial split (the ‘root node’) that maximizes the separation of the variable of interest (in this case, RT-PCR positive or negative), then the next best split is identified (for ‘secondary nodes’), then the next, and so on. The tree was subsequently pruned using the ptree package, applying the complexity parameter that was found to minimize cross-validated error.

An initial training set was used to build the model, using data from the years 2009–2013. The model was subsequently applied to a test set of data from 2014. Measures of model performance were calculated by comparing the predicted outcome of RT-PCR (positive or negative for influenza) against the observed result. Performance measures for the QuickVue test were calculated using the RT-PCR result for influenza as the gold standard for comparison. All analyses were performed using IBM SPSS Statistics version 23.0 (IBM Corp., Armonk, NY) and R version 3.3.0 (R Core Team, Vienna, Austria).

## Results

A total of 4572 individuals were enrolled between August 2009 and August 2014, of which 1493 (32.7%) had detectable influenza RNA by RT-PCR (hereafter described as influenza “cases”). The majority of influenza cases were attributable to influenza B (38.6%), then influenza A(H1N1)pdm09 (35.1%), followed by influenza A(H3N2) (26.4%). All influenza A infections were attributable to influenza A(H1N1)pdm09 or influenza A(H3N2) in the cohort. There was one case of dual infection with influenza B and influenza A(H1N1)pdm09 detected by RT-PCR.

### Comparison of influenza-positive and influenza-negative cases (Table 1)

Influenza cases were significantly older than non-influenza ILI cases, with mean ages of 14.8 and 8.7 years, respectively (p<0.01 by t-test, data not shown). 46.5% of ILI cases occurred in children less than 4 years of age, however, only 15.5% of ILI cases in young children were influenza positive by RT-PCR versus 49.0% of individuals aged 15–59 years. Individuals with ILI and no comorbidities were more likely to test positive for influenza infection by RT-PCR (33.2%) than those with one or more comorbidity (27.4%). Outpatients with ILI were more likely to test positive for influenza infection than inpatients (33.6% versus 19.2%). Healthcare workers presenting with ILI were significantly less likely to be influenza-positive (38.2%), compared with homemakers (63.5%) and members of the military (53.6%). The proportion of ILI cases that were influenza positive by RT-PCR varied significantly by year, with maximum positivity in 2010 (42.8%) and minimum positivity in 2013 (18.4%).

**Table 1:** Predictors of influenza positivity among patients presenting with influenza-like illness, predictors of reported vaccination within the last 12 months, and vaccine efficacy.

		Total	Flu + (%)	P^a^	% Vaccinated	P	VE (95% CI)
**Overall**		4572	(1493) 32.7%	—	21.0%	—	49.5% (40.0–57.1%)
**Subtype**^**b**^	A(H3N2)	392	26.4%	—	17.0%	<0.01	36.2% (15.9–51.5%)
	A(H1N1)pdm09	524	35.1%	—	9.7%		66.6% (54.9–75.3%)
	Flu B	257	38.6%	—	16.0%		41.1% (25.3–53.6%)
**Age (years)**	0–4	2126	15.5%	<0.01	27.0%	<0.01	24.5% (0.01–43.2%)
	5–14	1433	46.5%		19.1%		40.1% (21.8–54.3%)
	15–59	950	49.0%		9.8%		56.7% (32.1–72.9%)
	60 +	63	48.6%		19.0%		51.9% (-73.0–88.4%)
**Gender**	Female	2489	31.5%	0.169	22.5%	0.028	50.1% (36.5–61.1%)
	Male	2083	33.5%		19.8%		48.1% (34.9–58.9%)
**Comobidities**^**c**^							
	Renal failure	35	34.3%	0.980	17.1%	0.723	
	Heart disease	51	23.5%	0.212	35.3%	**0.019**	
	COPD	13	0.0%	**0.026**	69.2%	**<0.01**	
	Asthma	157	29.3%	0.401	42.7%	**<0.01**	37.2% (-26.7–69.7%)
	Obesity	2	50.0%	1.000	50.0%	0.889	
	Pregnancy	0	—	—	—	—-	
	Diabetes	9	22.2%	0.755	11.1%	0.750	
	Immunocompromised	8	37.5%	1.000	50.0%	0.114	
	Hematologic	176	27.8%	0.19	25.6%	0.154	52.5% (-6.5%-80.9%)
	Liver disease	2	0.00%	1.000	0.00%	1.000	
**# Comorbid**	None	4121	33.2%		19.6%		49.3% (39.4–57.8%)
**Conditions**	1	449	27.4%	**0.038**	33.6%	**<0.01**	42.2% (0.09–64.1%)
	2	2	50.0%		0.00%		—
**Ward**	Inpatient	287	19.2%	**<0.01**	22.6%	0.526	38.5% (-28.2–73.2%)
	Outpatient	4285	33.6%		20.9%		49.6% (40.2–57.6%)
**Occupation**	Healthcare	183	38.2%	**<0.01**	22.4%	**<0.01**	79.1% (50.5–92.5%)
	Homemaker	52	63.5%		11.5%		-17.2% (-810–79.4%)
	Military	194	53.6%		9.3%		85.1% (53.0–96.6%)
	Office	263	49.4%		6.8%		19.3% (-111–70.1%)
	University	248	49.2%		5.6%		37.2% (20.9–50.4%)
**Year**	2009	237	19.4%		16.0%		41.6% (-47.1–80.9%)
	2010	1352	42.8%	**<0.01**	12.6%	**<0.01**	46.9% (25.3–62.8%)
	2011	798	27.3%		26.1%		49.1% (25.3–66.0%)
	2012	802	35.5%		20.1%		37.6% (9.2%–57.7%)
	2013	593	18.4%		21.9%		17.9% (-36.1–52.3%)
	2014	790	32.4%		32.0%		59.1% (42.2–71.5%)

^a^ P-values were calculated using Mantel-Haenszel χ^2^ statistics, comparing numbers of individuals with and without influenza, or with and without a history of vaccination, across strata for each variable of interest. For comorbidities, χ^2^ statistics were based upon the presence or absence of each condition (e.g., numbers of individuals with influenza, among those with and without renal disease). Exact testing was performed for comparisons with cells with values < = 5. Comparisons that were statistically significantly with α = 0.05 are shown in **bold**.

^b^ For influenza subtypes, data are displayed as column percents (i.e., the percent of influenza isolates that were influenza A(H1N1)pdm09, influenza A(H3N2), and influenza B viruses, respectively). For the remaining categories, row percents are displayed (i.e., the percent of specimens for each stratum that was influenza).

^c^ Comorbidities listed are those recommended by the US CDC to receive influenza vaccine. VE was not calculable for all strata due to low numbers.

### Vaccination history and vaccine effectiveness (Table 1)

21% (960 / 3612) of individuals with ILI reported a history of influenza vaccination in the last 12 months, with an associated overall vaccine effectiveness of 49.5% (95% confidence interval [CI] 40.0–57.1%). VE was highest for influenza A(H1N1)pdm09 (66.6%) and lowest for influenza A(H3N2) (36.2%). Young children were most likely to have been vaccinated within the last 12 months (27.0%) but VE was lowest in this age group at 24.5%. Individuals aged 15–59 years were least likely to report vaccination (9.8%) but VE was highest in this age group at 56.7%. 33.6% of those with any underlying comorbidity reported influenza vaccination, compared to 19.6% of those with none. Vaccination history varied by occupation, with healthcare workers the most likely to be vaccinated (22.4%) and office workers, university students, and members of the military the least. Notably, VE was very high in both healthcare workers (79.1%) and members of the military (85.1%). Likelihood of vaccination increased from 16.0% in 2009 to 32.0% in 2014. VE varied significantly by year, from a minimum of 17.9% in 2013 to a maximum of 59.1% in 2014.

### Temporal trends in ILI cases

**Fig 1:**
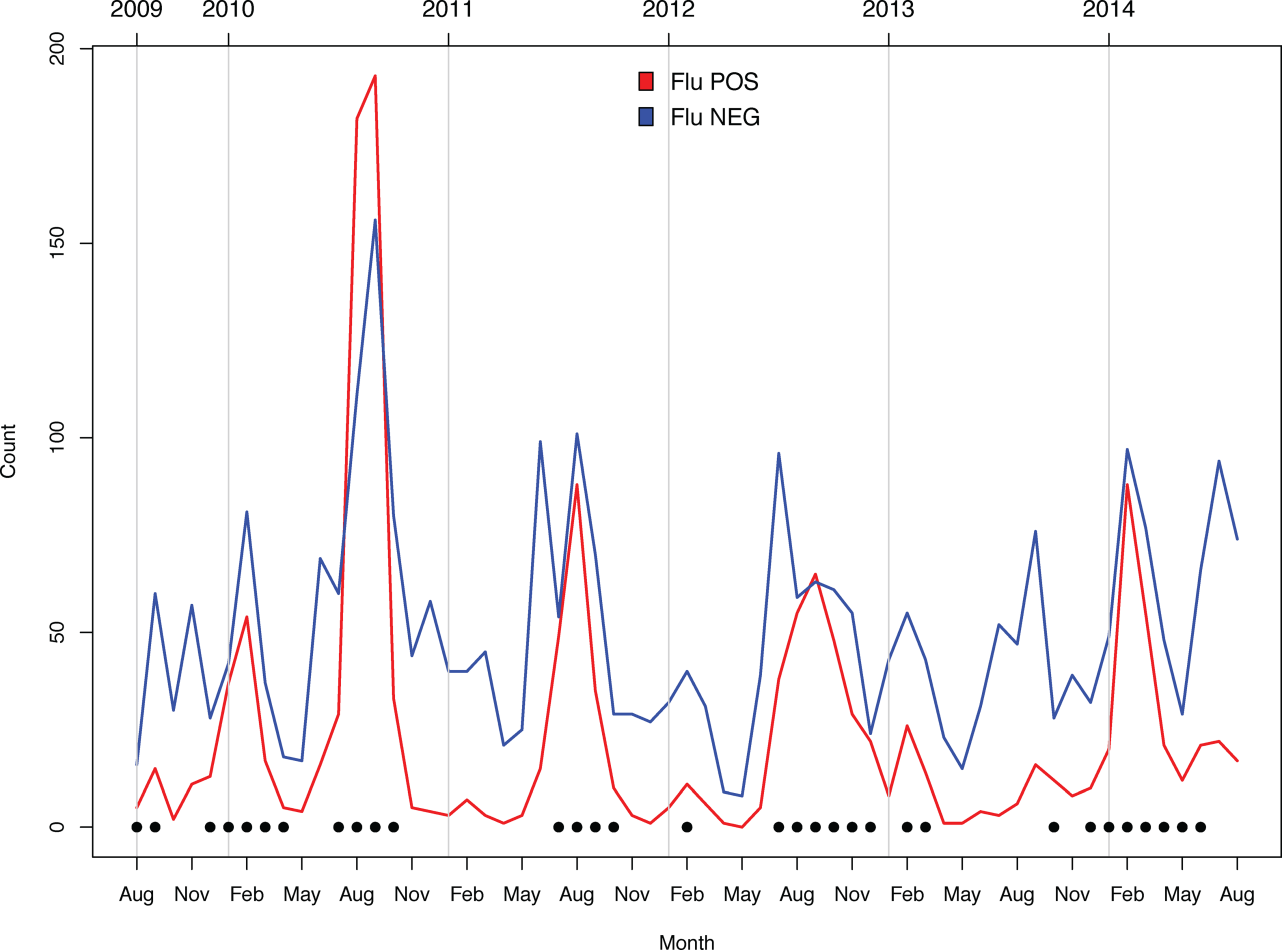
Temporal distribution of ILI cases testing positive for influenza infection (RED) and negative for influenza infection (BLUE). Solid circles indicate months identified as experiencing influenza outbreaks (defined as months wherein > = 25% of ILI specimens tested positive for influenza infection by RT-PCR).

The seasonality of influenza cases roughly followed a biphasic pattern, with the largest peak often occurring between July and September and a smaller peak often occurring between January and March (Fig 1). An exception to this pattern was late 2012 –early 2014, where the pattern varied unpredictably and for reasons which remain unclear. In contrast to the biphasic peaks of influenza activity, the detection of influenza-negative ILI cases followed no discernable pattern and tended to occur year-round.

**Fig 2:**
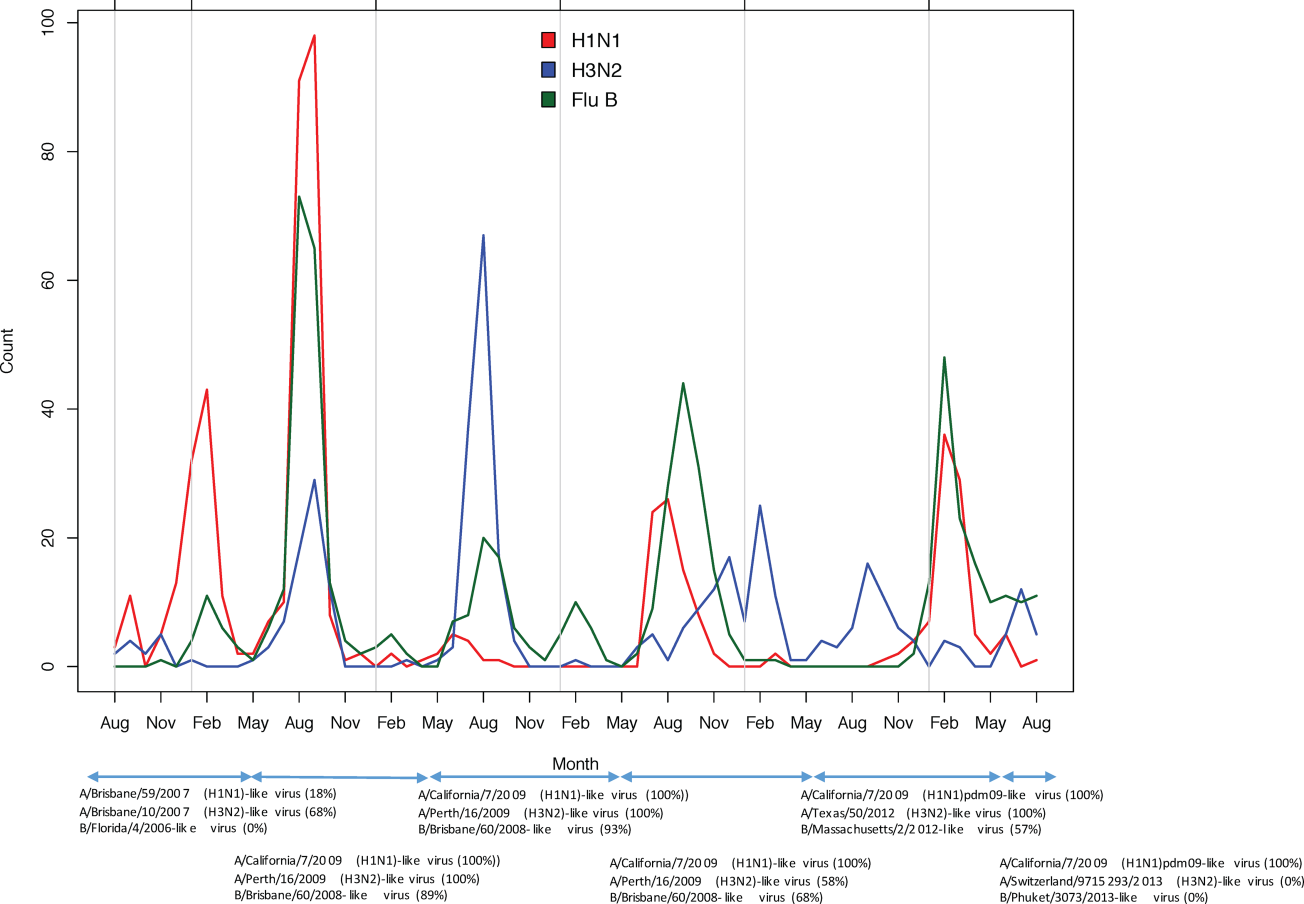
Temporal distribution of subtypes, with southern hemisphere vaccine composition by year. The percent of influenza A(H1N1)pdm09, influenza A(H3N2), and influenza B isolates matching the vaccine strain for each year is shown in parentheses [23]. Note that the Southern hemisphere vaccine typically became available in May of each year.

A large increase in influenza cases in 2010 was largely attributable to influenza A(H1N1)pdm09 (Fig 2) and 100% of H1N1 strains detected from 2010–2014 were A(H1N1)pdm09. Notably, there was a mismatch in the influenza B component of the vaccine in 2009 and a large influenza B outbreak in 2010. Subsequently, the influenza B vaccine strain represented 68%, 57%, and 0% of detected influenza B strains in 2012, 2013, and 2014, respectively. The vaccine strain of influenza A(H3N2) was changed several times during the study period and represented between 0 and 100% of detected influenza A(H3N2) strains during the study period 2009–2014. No distinct differences in seasonality were observed between subtypes.

### Clinical predictors (Table 2)

3.7% of influenza cases were hospitalized. Hospitalization was most common among young children with influenza infection (8.8%) and least common among those aged 15–59 years (0.9%). The rate of hospitalization among influenza cases varied significantly by year, from a minimum of 0.7% in 2012 to a maximum of 4.3% in 2014. 25.4% of inpatients with influenza infection received oseltamivir at some point prior to enrollment versus 1.0% of outpatients. Oseltamivir administration prior to enrollment was most common in the youngest and oldest age groups. Individuals with underlying comorbidities were more likely to be hospitalized (8.9% versus 3.2%) compared to those without comorbidities. 22.4% of influenza cases reported having received antibiotics at some point prior to enrollment; reported rates of antibiotic administration were higher in individuals aged 0–4 years and greater than 60 years and for inpatients.

**Table 2:** Predictors of clinical severity and receipt of antimicrobial medications among those with PCR-confirmed influenza infection.

		Total	% IPD	p^a^	% Oseltamivir	P
**Overall**	Inpatient	55	3.7%	—	25.4%	**<0.001**
	Outpatient	1438	—		1.0%	
**Subtype**	A(H3N2)	392	4.1%		3.6%	
	A(H1N1)pdm09	524	3.4%	0.847	1.1%	0.052
	Flu B	576	3.6%		1.6%	
**Age (years)**	0–4	329	8.8%		2.7%	
	5–14	667	3.1%	**<0.001**	2.5%	**0.019**
	15–60	467	0.9%		0.4%	
	60 +	30	3.3%		3.3%	
**Gender**	Female	658	4.4%	0.238	1.8%	0.917
	Male	835	3.1%		2.0%	
**# Comorbid**	None	1369	3.2%		1.9%	
**conditions**	1	123	8.9%	**0.005**	2.4%	0.755
	2	1	0.0%		0.0%	
**Occupation**	Healthcare	70	0%		0%	
	Homemaker	33	0%	0.052	0%	0.171
	Military	104	0%		0%	
	Office	130	0%		0%	
	University	38	3.3%		1.7%	
**Year**	2009	46	4.3%		2.2%	
	2010	579	5.5%	**0.010**	1.2%	0.092
	2011	218	1.8%		2.3%	
	2012	285	0.7%		1.4%	
	2013	109	3.7%		5.5%	
	2014	256	4.3%		2.3%	

^a^ P-values were calculated using Mantel-Haenszel χ^2^ statistics, comparing numbers of individuals recruited in inpatient or outpatient departments, or with and without a history of oseltamivir, across strata for each variable of interest. Exact testing was performed for comparisons with cells with values < = 5. Comparisons that were statistically significantly with α = 0.05 are shown in **bold**.

Among ILI cases, individuals who were influenza-positive were more likely to have had fever at enrollment and more likely to report cough, sore throat, chills, malaise, and generalized body aches (Supplemental table). They were less likely to have difficulty breathing, diarrhea, and lung findings on exam than non-influenza ILI cases. Individuals with influenza A(H3N2) infection were more likely to report fever, runny, nose, or malaise at enrollment and less likely to report cough.

### CART analysis of symptoms predicting influenza RT-PCR positive and negativity

**Fig 3:**
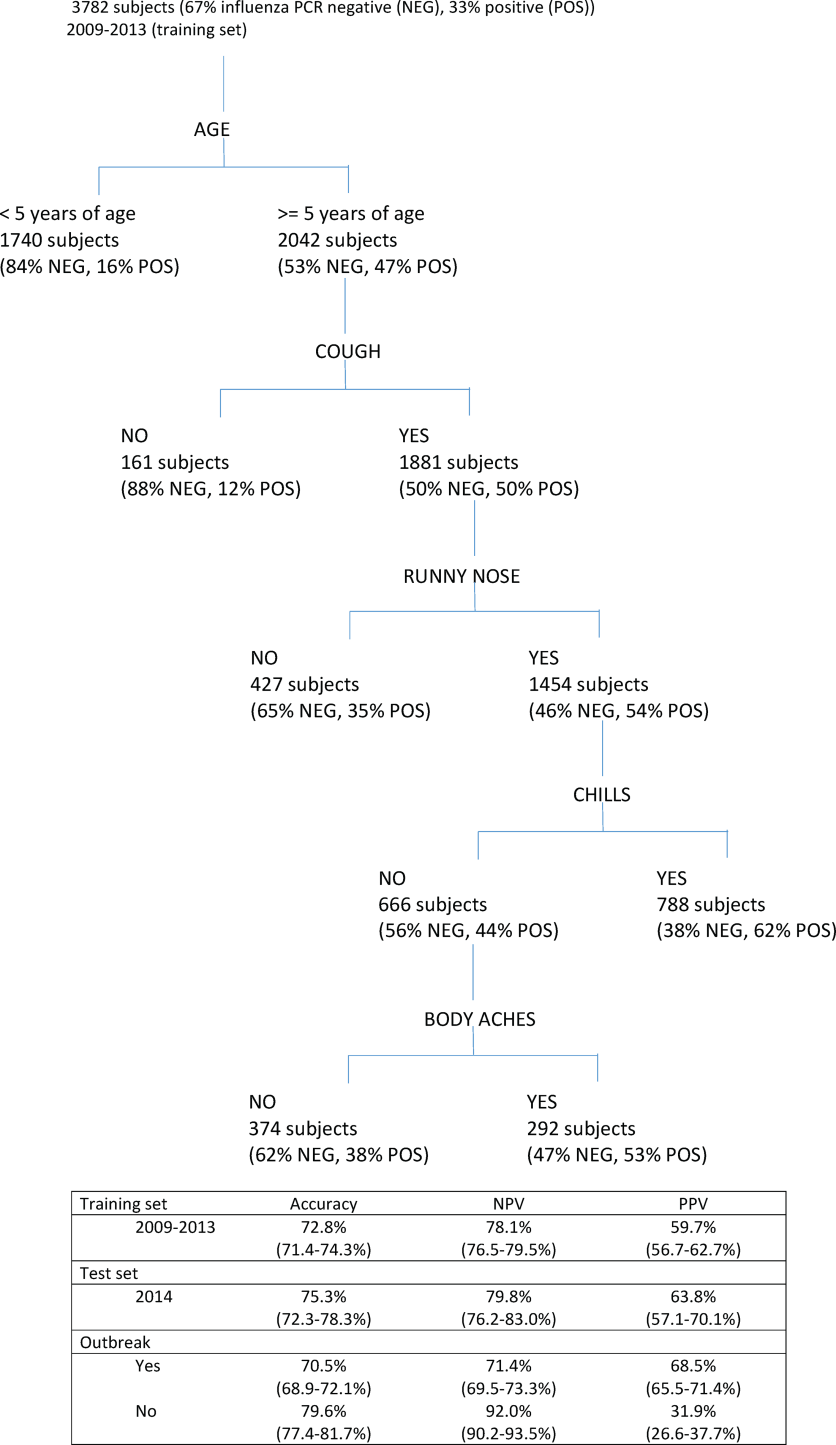
CART analysis to predict influenza RT-PCR positivity on the basis of clinical symptoms.

Age was the first cut-point identified by the recursive partitioning algorithm, with 84% of children less than 5 years of age with ILI testing negative by influenza RT-PCR (Fig 3). Cough was the next cut-point assigned to children older than 5 years, with 88% of those lacking cough testing negative by RT-PCR. Among those with cough, 65% of those without runny nose tested negative by RT-PCR. Among those with cough and runny nose, 62% of those with chills tested positive for influenza infection by RT-PCR, while those without chills were further divided by the presence or absence of body aches. 62% of those without chills or body aches tested negative by RT-PCR.

**Fig 4:**
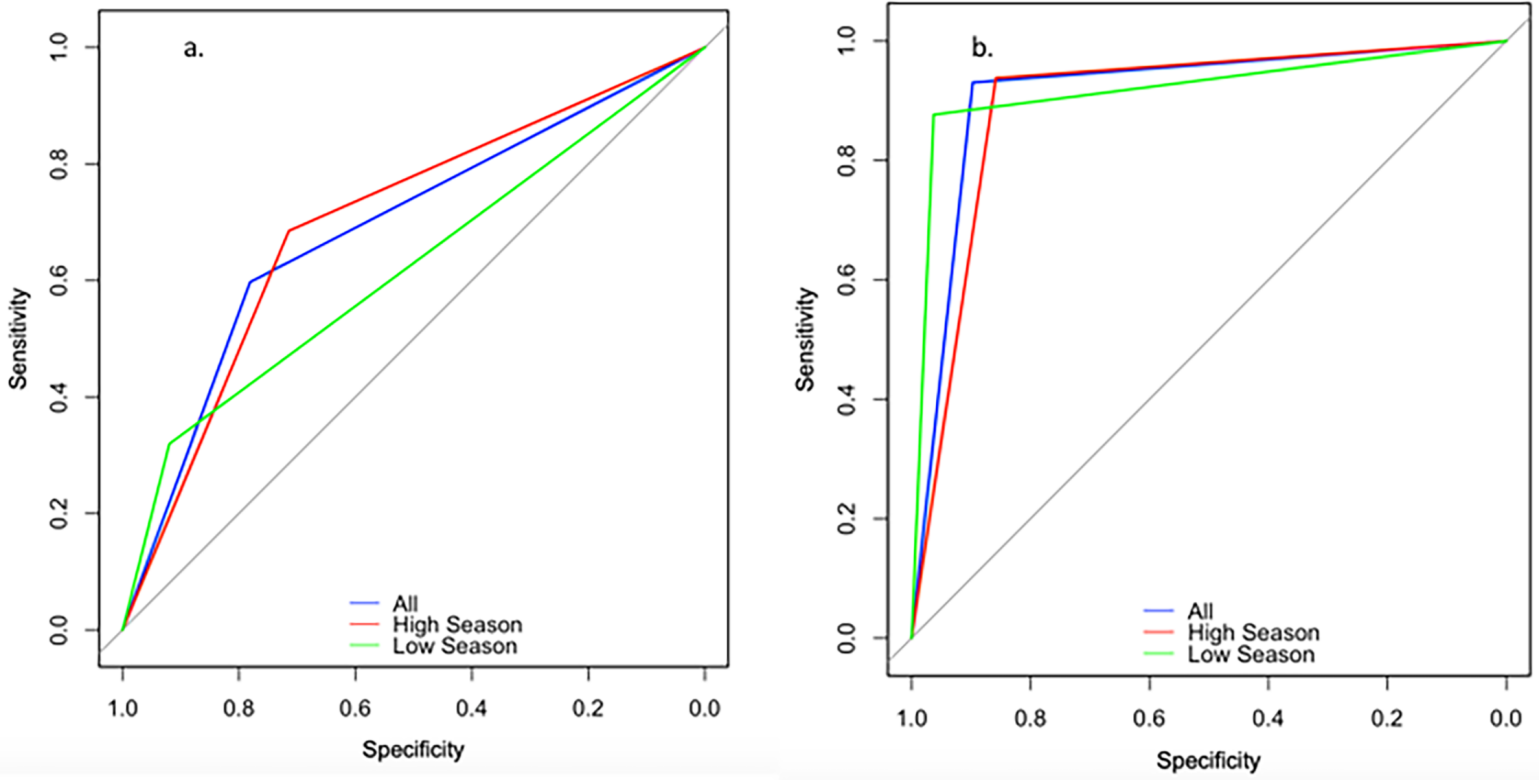
Receiver operating characteristic (ROC) curves for (Fig 3A) CART analysis and (Fig 3B) QuickVue, compared to RT-PCR for identifying influenza infection among individuals with ILI.

The overall accuracy of the model based upon the training set (data limited to years 2009–2013) was 72.8%. The specificity and negative predictive value (NPV) of the model were superior to the sensitivity and positive predictive value (PPV) for the model, at 82.9% and 78.4% versus 52.1% and 59.7%. When applied to the test set (data from 2014), the accuracy for correctly predicting influenza RT-PCR result based upon symptom data was similar (75.3%). Notably, PPV doubled when the algorithm was applied to epidemic periods (68.5% versus 31.9% for non-epidemic periods). Conversely, NPV was highest during non-epidemic periods (92.0% versus 71.4% for epidemic periods). The receiver operating characteristic (ROC) curves for the CART analysis overall, epidemic, and non-epidemic periods are shown in Fig 4A. Area under the curve (AUC) was 0.689 overall, 0.619 for non-epidemic periods, and 0.700 for epidemic periods.

### Sensitivity and specificity of QuickVue (Table 3)

The overall sensitivity of the QuickVue test was 77.0%, specificity was 97.2%, NPV 89.7%, and PPV 93.0%. The sensitivity of the QuickVue test was higher for influenza B than for influenza A viruses (80.6% versus 73.4%); specificity was high for both at >98%. 16 specimens were positive for both influenza A and influenza B viruses by QuickVue testing; 11 (68.8%) of these subsequently tested negative by RT-PCR (data not shown). The performance of the QuickVue test was not notably different by inpatient versus outpatient status. The sensitivity of QuickVue was 62.9% if performed on the day of illness onset and peaked at 2 days post-onset of symptoms (79.8%). Sensitivity was lowest in those aged 15–59 years of age (72.8%) and highest at the extremes of age. Sensitivity was lower in those with a history of oseltamivir administration (65.5%) compared to those without a history of oseltamivir (77.2%). PPV was highest during epidemic periods (93.7%) compared to non-epidemic periods (87.7%). The ROC curves for QuickVue overall, epidemic, and non-epidemic periods are shown in Fig 4B. Area under the curve (AUC) was 0.914 overall, 0.920 for non-epidemic periods, and 0.898 for epidemic periods.

**Table 3:** QuickView test performance, as compared to RT-PCR (“gold standard”). Lower limit of 95% confidence interval for each proportion is shown in parentheses.

		*Sens*	*Spec*	*NPV*	*PPV*
Overall		77.0% (74.8%)	97.2% (96.6%)	89.7% (88.6%)	93.0% (91.5%)
Subtype					
	Flu A	73.4% (70.8%)	98.4% (97.9%)	93.7% (92.9%)	91.9% (89.6%)
	Flu B	80.6% (77.1%)	98.7% (98.3%)	97.2% (96.7%)	90.3% (87.4%)
Outbreak					
	Yes	77.4% (75.0%)	96.4% (95.4%)	85.8% (84.2%)	93.8% (92.2%)
	No	74.5% (97.6%)	98.4% (97.5%)	96.3% (95.1%)	87.7% (81.4%)
Disposition					
	Inpatient	74.5% (61.0%)	98.7% (96.2%)	93.2% (90.5%)	94.2% (81.3%)
	Outpatient	77.1% (74.9%)	97.1% (96.4%)	89.4% (88.2%)	93.0% (91.4%)
Days since onset					
	0	62.9% (49.7%)	98.8% (95.7%)	87.7% (82.1%)	95.1% (83.5%)
	1	78.5% (75.2%)	97.1% (96.0%)	88.8% (87.0%)	93.9% (91.6%)
	2	79.8% (76.0%)	97.3% (96.1%)	91.1% (89.3%)	93.3% (90.5%)
	3	72.0% (65.8%)	96.5% (94.7%)	89.3% (86.6%)	89.6% (84.3%)
	4+	64.3% (35.1%)	100.0% (94.0%)	92.3% (82.9%)	100.0% (66.4%)
Age					
	0–4	80.2% (75.5%)	97.3% (96.5%)	96.4% (95.5%)	84.9% (80.4%)
	5–14	78.0% (74.6%)	96.1% (94.4%)	83.3% (80.7%)	94.5% (92.3%)
	15–59	72.8% (68.5%)	98.1% (96.4%)	78.9% (75.3%)	97.4% (95.2%)
	>60	86.7% (69.3%)	100.0% (89.4%)	89.2% (74.6%)	100.0% (86.8%)
Oseltamivir					
	Yes	65.5% (45.7%)	95.7% (85.5%)	81.8% (69.1%)	90.5% (69.6%)
	No	77.2% (75.0%)	97.2% (96.6%)	89.8% (88.7%)	93.1% (91.5%)

## Discussion

Our clinical prediction algorithm to discern influenza from non-influenza ILI had moderate accuracy (73%) but poor PPV (59.7%). This is consistent with prior studies which indicated that it is more feasible to identify what is not influenza than what is influenza among patients with ILI [14–17], given significant overlap of clinical symptoms between influenza and other respiratory pathogens. PPV improved to 69% during epidemic months, however, overall test performance (by AUC) was not dramatically improved. For this study, our efforts were likely further limited by a lack of longitudinal data regarding the time course of symptoms. Future efforts will include retrospective chart reviews of influenza cases and hospitalized patients to inform the development of more dynamic clinical prediction algorithms and to attempt to identify individuals at high risk of progression to severe disease.

Sensitivity of the QuickVue rapid test was >70% overall for both influenza A and B viruses, superior to prior studies reporting sensitivity in ambulatory populations of approximately 20–55% [24–26]. In our study, the timing of rapid test administration was found to be an important predictor of test performance, with peak sensitivity 1–2 days after the onset of symptoms, likely due to the dynamics of viral shedding in the nasopharynx after infection. As for the CART analysis, PPV improved during epidemic periods with a higher proportion of influenza-positive ILI cases. The lower sensitivity of the QuickVue test for adults, inpatients, and individuals who had previously received oseltamivir should serve as a caution to providers that a negative test result should not be interpreted to mean that influenza infection has been ruled out or to indicate that oseltamivir should not be given or continued.

Early treatment with neuraminidase inhibitors is recommended in individuals hospitalized with influenza and individuals at high risk of progression to severe disease. Antiviral coverage was low in the study population, though notably this was a cross-sectional study and individuals may have received oseltamivir later in their treatment course. There was a relatively high rate of reported antimicrobial administration (22%), which did not differ significantly among individuals with PCR-confirmed influenza infection by the presence or absence of an infiltrate on chest X-ray, by lung findings on physical exam, or by the presence or absence of comorbidities that may predispose to severe disease (data not shown). The high rate of antibiotic use and low rate of oseltamivir administration prior to study enrollment in this population underscore the need for early and enhanced diagnosis of influenza influenza to facilitate patient triage and the appropriate targeting of antimicrobials.

Influenza vaccine coverage has been increasing in recent years in the study population but remains relatively low at 20–30%. Vaccine effectiveness varied by year but was moderate overall at 49.5%, consistent with an earlier study in this cohort [27]. Notably, we report a VE in young children of 24%, which is much lower than the 55–60% estimated previously [28]. This may be explained by a disproportionate burden of influenza A(H3N2) infection in young children in our study (data not shown), as influenza A(H3N2) not well matched to the vaccine strain for some years. Vaccine coverage in those with relevant comorbidities was low at 33.0%. Further, vaccine coverage was low in members of the military and healthcare workers, at 22% and 9%, respectively, despite high VE (>75%) in both groups. These findings indicate that there are populations of adults within the study population that could greatly benefit from targeted vaccination campaigns, to include military and healthcare professionals and individuals with comorbidities. Studies of vaccine acceptance among adult patients and medical providers would provide insight into reasons for this low uptake and indicate areas for targeted intervention.

It is generally thought that the influenza vaccine becomes significantly protective after two weeks, with efficacy waning considerably in the months following administration [29, 30]. The timing and number of peaks of influenza activity varied unpredictably in our study population, which complicates timing of vaccine delivery to optimize VE and, more broadly, selection of the northern versus southern hemisphere vaccine strains. Future studies should consider the potential for climatological data and prior influenza epidemic data to possibly predict these peaks in activity and inform vaccination programs.

These analyses were subject to multiple limitations. Foremost is that patient assessment was limited to a single point in time, precluding analysis of how symptoms, severity, and clinical management may have changed over time. Patient treatment and vaccination histories were self-reported and possibly subject to recall bias. This study was conducted at a single hospital in Thailand and may have limited generalizability beyond the Asia-Pacific region. A five-year period may be insufficient to assess patterns of seasonality and vaccine effectiveness, particularly given that 2009–2010 were unique years due to the dramatic emergence of influenza A(H1N1)pdm09 and the introduction of the swine flu vaccine. This study is intended to continue for ten years, which will allow assessment of patterns of influenza transmission and vaccine performance over a longer interval.

## Conclusions

Despite the widespread availability of influenza vaccines and antivirals, the global burden of influenza-related disease continues to be high. In this manuscript, we report on predictors of vaccination, influenza infection, and vaccine effectiveness in a cohort of individuals presenting with ILI to an urban Thai hospital. Based upon the significant overlap in the clinical presentation between influenza and other influenza-like illnesses, we do not recommend the routine use of clinical algorithms for the identification of seasonal influenza. We suggest that the expanded use of rapid influenza diagnostic tests such as QuickVue would allow early detection of influenza infection in resource-limited settings and facilitate the appropriate allocation of antimicrobial medications. Finally, we suggest that there are multiple groups who would benefit from targeted vaccination campaigns such as members of the military, healthcare workers, and adults with medical comorbidities, given their relatively high rates of influenza infection but low rates of reported vaccination.

## Supporting information

S1 Table Clinical presentation of influenza positive and influenza negative cases. ^a^ P-values were calculated using Mantel-Haenszel χ^2^ statistics, with exact testing performed for comparisons with cells with values < = 5. Comparisons that were statistically significantly with α = 0.05 are shown in bold.
(DOCX)
