# Supplementary material for: Clinical and laboratory predictors of influenza infection among individuals with influenza-like illness presenting to an urban Thai hospital over a five-year period
Source: PLoS One. 2018 Mar 7;13(3):e0193050. doi: 10.1371/journal.pone.0193050 (PMC5841736; doi:10.1371/journal.pone.0193050)
Supplement: S1 Table — a P-values were calculated using Mantel-Haenszel χ2 statistics, with exact testing performed for comparisons with cells with values < = 5. Comparisons that were statistically significantly with α = 0.05 are shown in bold. (DOCX) [file pone.0193050.s001.docx]

| **S1 Table. Clinical presentation of influenza positive and influenza negative cases** | | | | |
| --- | --- | --- | --- | --- |
|  |  | Flu -  (Column %) | Flu +  (Column %) | p-value^a^ |
|  |  |  |  |  |
| Disposition |  |  |  |  |
|  | *Inpatient* | 232  (7.5%) | 55  (3.7%) | <0.001 |
|  | *Outpatient* | 2847  (92.5%) | 1438  (96.3%) |  |
| Fever at time of presentation | | |  |  |
|  | *No* | 916  (29.7%) | 310  (20.8%) | <0.001 |
|  | *Yes* | 2162  (70.2%) | 1183  (79.2%) |  |
| Cough | | | | |
|  | *No* | 296  (9.6%) | 39  (2.6%) | <0.001 |
|  | *Yes* | 2782  (90.4%) | 1451  (97.4%) |  |
| Sore throat | | | | |
|  | *No* | 804  (28.5%) | 367  (25.1%) | <0.001 |
|  | *Yes* | 2016  (71.5%) | 1093  (74.5%) |  |
| Difficulty breathing | | | | |
|  | *No* | 1483  (48.4%) | 855  (57.4%) | <0.001 |
|  | *Yes* | 2782  (51.6%) | 635  (42.6%) |  |
| Runny nose | | | | |
|  | *No* | 638  (20.8%) | 277  (15.2%) | <0.001 |
|  | *Yes* | 2433  (79.2%) | 1264  (84.8%) |  |
| Chills | | | | |
|  | *No* | 2090  (68.2%) | 693  (46.5%) | <0.001 |
|  | *Yes* | 974  (31.8%) | 797  (53.5%) |  |
| Malaise | | | | |
|  | *No* | 1267  (41.7%) | 506  (34.0%) | <0.001 |
|  | *Yes* | 1770  (58.3%) | 983  (66.0%) |  |
| Generalized muscle / joint pain | | | | |
|  | *No* | 870  (47.0%) | 427  (33.1%) | <0.001 |
|  | *Yes* | 980  (53.0%) | 862  (66.9%) |  |
| Diarrhea | | | | |
|  | *No* | 2631  (85.7%) | 1320  (88.5%) | 0.008 |
|  | *Yes* | 438  (14.3%) | 171  (11.5%) |  |
| Lung findings on exam | | | | |
|  | *No* | 2378  (78.8%) | 1191  (81.5%) | 0.036 |
|  | *Yes* | 638  (21.1%) | 270  (18.5%) |  |

^a^ P-values were calculated using Mantel-Haenszel χ^2^ statistics, with exact testing performed for comparisons with cells with values <=5. Comparisons that were statistically significantly with α = 0.05 are shown in **bold**.
